# Author Correction: Quantum-anomalous-Hall current patterns and interference in thin slabs of chiral topological superconductors

**DOI:** 10.1038/s41598-024-52468-8

**Published:** 2024-01-23

**Authors:** Daniele Di Miceli, Llorenç Serra

**Affiliations:** 1 Institute for Cross-Disciplinary Physics and Complex Systems IFISC (CSIC-UIB), 07122 Palma, Spain; 2 Department of Physics and Materials Science, University of Luxembourg, 1511 Luxembourg, Luxembourg; 3 Department of Physics, University of the Balearic Islands, 07122 Palma, Spain

Correction to: *Scientific Reports* 10.1038/s41598-023-47286-3, published online 15 November 2023

The Acknowledgements section in the original version of this Article was incomplete.

“This project is financially supported by the QuantERA grant MAGMA by MCIN/AEI/10.13039/501100011033 under project PCI2022-132927. L.S. acknowledges support from Grants No. PID2020-117347GB-I00 funded by MCIN/AEI/10.13039/501100011033 and No. PDR2020-12 funded by GOIB.”

now reads:

“This project is financially supported by the QuantERA grant MAGMA by MCIN/AEI/10.13039/501100011033 and by European Union NextGenerationEU/PRTR under project PCI2022-132927. L.S. acknowledges support from Grants No. PID2020-117347GBI00 funded by MCIN/AEI/10.13039/501100011033 and No. PDR2020-12 funded by GOIB.”

The original Article has been corrected.

